# Assessment of the uncertainty and interpretability of deep learning models for mapping soil salinity using DeepQuantreg and game theory

**DOI:** 10.1038/s41598-022-19357-4

**Published:** 2022-09-07

**Authors:** Aliakbar Mohammadifar, Hamid Gholami, Shahram Golzari

**Affiliations:** 1 Department of Natural Resources Engineering, University of Hormozgan, Bandar-Abbas, Hormozgan Iran; 2 Department of Electrical and Computer Engineering, University of Hormozgan, Bandar-Abbas, Hormozgan Iran; 3 Deep Learning Research Group, University of Hormozgan, Bandar Abbas, Hormozgan Iran

**Keywords:** Environmental sciences, Natural hazards

## Abstract

This research introduces a new combined modelling approach for mapping soil salinity in the Minab plain in southern Iran. This study assessed the uncertainty (with 95% confidence limits) and interpretability of two deep learning (DL) models (deep boltzmann machine—DBM) and a one dimensional convolutional neural networks (1DCNN)—long short-term memory (LSTM) hybrid model (1DCNN-LSTM) for mapping soil salinity by applying DeepQuantreg and game theory (Shapely Additive exPlanations (SHAP) and permutation feature importance measure (PFIM)), respectively. Based on stepwise forward regression (SFR)—a technique for controlling factor selection, 18 of 47 potential controls were selected as effective factors. Inventory maps of soil salinity were generated based on 476 surface soil samples collected for measuring electrical conductivity (ECe). Based on Taylor diagrams, both DL models performed well (RMSE < 20%), but the 1DCNN-LSTM hybrid model performed slightly better than the DBM model. The uncertainty range associated with the ECe values predicted by both models estimated using DeepQuantilreg were similar (0–25 dS/m for the 1DCNN-LSTM hybrid model and 2–27 dS/m for DBM model). Based on the SFR and PFIM (permutation feature importance measure)—a measure in game theory, four controls (evaporation, sand content, precipitation and vertical distance to channel) were selected as the most important factors for soil salinity in the study area. The results of SHAP (Shapely Additive exPlanations)—the second measure used in game theory—suggested that five factors (evaporation, vertical distance to channel, sand content, cation exchange capacity (CEC) and digital elevation model (DEM)) have the strongest impact on model outputs. Overall, the methodology used in this study is recommend for applications in other regions for mapping environmental problems.

## Introduction

Soil salinization is a major desertification and land degradation process in arid and semi-arid areas. Globally, such degradation impacts one billion ha of land in many countries including China, India, Pakistan, Iran, Australia, and the United States^1^. Economically, saline soil causes a global loss in agricultural production equivalent to ~ 12.7–27.3 billion dollars per year since salinization can reduce food production by 50%^2^. Consequently, the monitoring and mapping of soil salinity is required to direct sustainable soil management.

Machine learning (ML)—a field of data science (DS)—provides useful tools for prediction purposes which can help us better understand the complex mechanisms controlling environmental phenomena^3,4^. ML algorithms can be divided into shallow and deep learning (DL). Typical shallow learning algorithms such as classification and regression tree (CART), bagged-CART (BCART), cforest (a type of random forest (RF)), support vector machine (SVM), quantile regression (QR) with LASSO penalty, ridge regression (RR), cubist, Bayesian additive regression trees (BART), extreme gradient boosting (XGB) and boosted regression trees (BTR) have been used to predict and model spatially different hazards such as gully erosion, wind erosion, water erosion, soil salinity, landslide susceptibility and dust emissions from land surfaces^5–7^.

DL has dramatically improved the state-of-art in many different artificial intelligence tasks including object detection, speech recognition and machine translation^8^. In comparison with shallow learning models, DL algorithms are especially promising tools for the spatial modelling of environmental hazards at various scales, due to the fact that the deeper networks designed by these models can address the weak fits commonly returned by shallow ML models^9^. For example, Gholami et al.^5^, reported successful applications of two DL models comprising restricted boltzmann machine (RBM) and simple recurrent neural network (RNN) for mapping land susceptibility to dust emissions. According to Mohammadifar et al.^10^, DL models (e.g., RNN-LSTM (long short-term memory), RNN-GRU (gated recurrent unit), DDNNs (dense deep neural networks) and DCNNs (deep convolutional neural networks)) performed better than shallow ML models (cforest, BCART, cubist, RR, SVM and QR-LASSO) for generating spatial maps of soil salinity in an arid catchment in southern Iran. Several other studies have reported that DL models, including deep learning-multilayer perceptions (DLMP)^11^, convolutional neural networks (CNN) and RNN^12^, deep neural networks (DNN)^13^ and deep learning neural network (DLNN)^14^ are useful for predicting evapotranspiration, flash floods and landslide susceptibility, respectively.

One area that DL model applications to date have not explored extensively is the uncertainty in predictions. To the best of our knowledge, this work is the first attempt at mapping soil salinity by combining stepwise forward regression (SFR) and DL model, and quantifying uncertainty associated with the DL models by DeepQuantreg—an efficient model. The current approach is general, and therefore, has high applicability for the spatial mapping of various environmental hazards worldwide. More specifically, the novelties of this study were: (1) feature selection by stepwise forward regression (SFR); (2) spatial mapping of soil salinity using two novel DL models comprising deep Boltzmann machine (DBM), and a one dimensional convolutional neural network (1DCNN)—long short-term memory (LSTM) hybrid model (1DCNN-LSTM hybrid model); (3) explicit quantification of the uncertainty associated with the predictions of the two DL models by applying DeepQuantreg, and; (4) assessment of the interpretability of the outputs from both DL models using game theory.

## Materials and methods

### Study area

**Figure 1:**
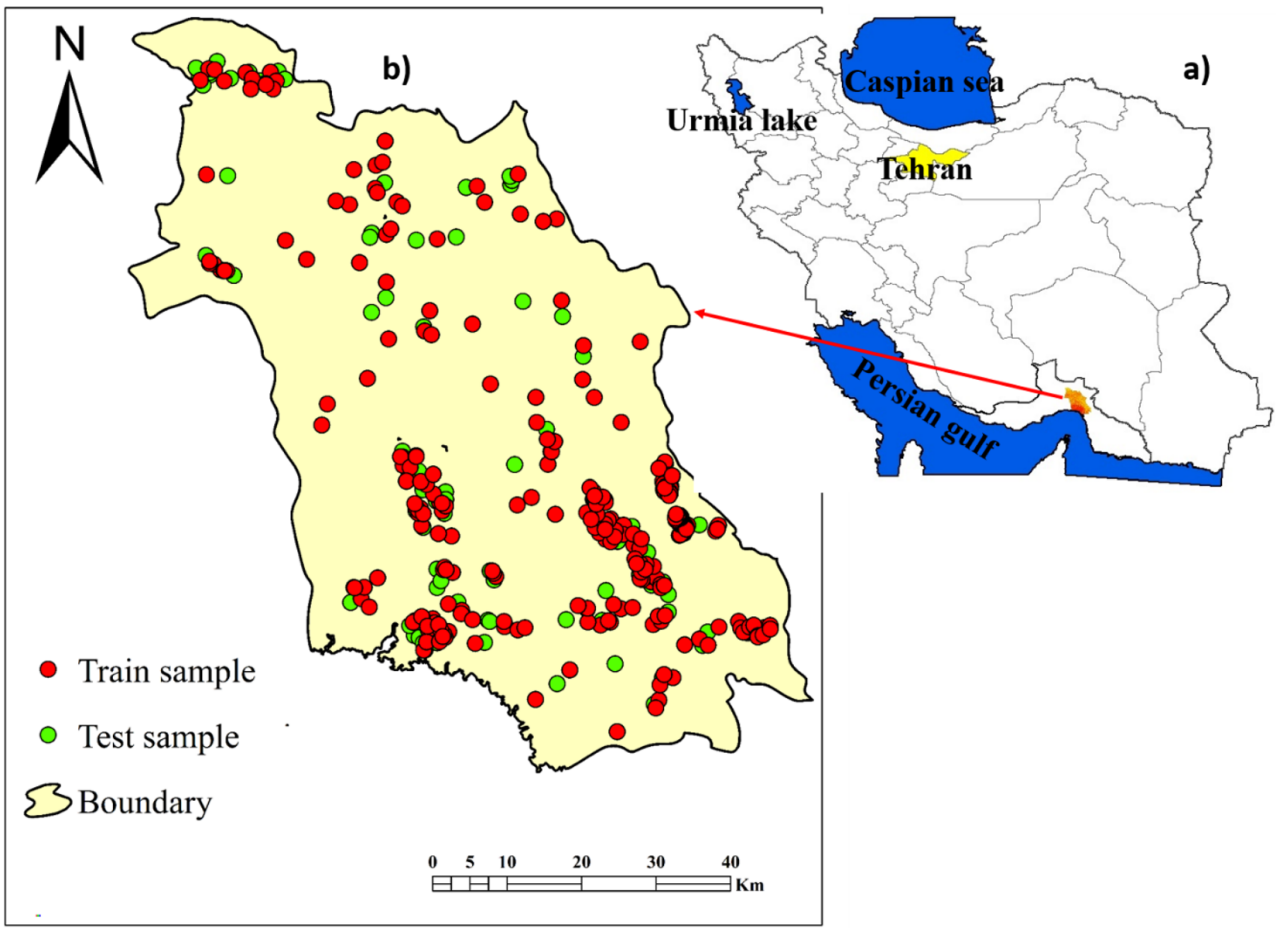
(**a**) Location of study area in Iran, and; (**b**) location of the soil sampling sites showing training and validation sample points.

The Minab plain (27° 01′ to 27° 56′ N and 50° 18′ to 51° 27′ E)—an arid region—is located in eastern Hormozgan province, southern Iran (Fig. 1). Elevations range between − 20 m (in southern and southeastern parts) to 3120 m (in northern parts). The Minab dam constructed in 1982 provides water for agricultural use and potable supplies for Bandar Abbas and Minab cities. Due to a high sediment rate (about 3.02 million m^3^/year during 1983–2005), the capacity of the reservoir is decreasing about 0.9% annually^15^. Based on data from the Minab meteorological station, mean annual precipitation ranges between 175 and 365 mm, and the mean annual temperature is 26.9 °C (between 2 and 49 °C). Groundwater pumping, mainly for agriculture use, during recent years has degraded groundwater quality due to saline water intrusion from southern area of study area—Persian Gulf. Therefore, the mapping of soil salinity is required to direct sustainable soil management.

### Factors controlling soil salinity and its inventory

Many environmental covariates potentially controlling soil salinity can be extracted from Landsat8 and DEM (digital elevation model) sources^7,16,17^. In this new study, we mapped 47 variables potentially controlling soil salinity consisting 15 variables related to the topography (e.g., aspect, curvature, DEM, flow direction, FSEN, length-slope factor (LSF), plan curvature, profile curvature, slope, terrain ruggedness index (TRI), terrain surface convexity (TSC), terrain surface texture (TST), topographic position index (TPI), topographic wetness index (TWI) and vertical distance to channel (VD)), 20 remote sensing-based variables (e.g., brightness index (BI), canopy response salinity index (CRSI), enhanced vegetation index (EVI), extended normalized difference vegetation index (ENDVI), generalized difference vegetation (GFV), global vegetation moisture index (GVMI), gypsum index, modified normalized difference water index (MNDWI), normalized difference vegetation index (NDVI), normalized difference infrared (NDI), salinity index (SI), salinity index (SI1), salinity index (SI2), salinity index (SI3), salinity index (SIB), salinity ration index (SRI), soil adjusted vegetation index (SAVI), two-band enhanced vegetation index (TEVI), world view improved vegetation index (WVIVI) and world view water index (WVWI)), eight soil characteristics (e.g., bulk density, cation exchange capacity (CEC), clay content, clay index, coarse fragment, organic carbon density (COD), sand content and silt content), two climatic variables (e.g., evaporation and precipitation), and two other variables (e.g., landuse and lithology) (Table 1). For generating the topographic-based variables, the DEM was downloaded from the https://earthexplorer.usgs.gov website at a spatial resolution of 30 m × 30 m. Finally, the variables were prepared based on the DEM by ArcGIS 10.7 and SAGA GIS. For producing the remote sensing-based variables, the Landsat 8 images were downloaded from the https://earthexplorer.usgs.gov for 10/04/2020, and then, transformed into a mosaic in the ENVI5.3 software. Generally, all these variables were produced at a spatial resolution of 30 m × 30 m. In this study, eight soil characteristics for the study area were downloaded from the https://soilgrids.org and then, these layers were georeferenced and mapped as a mosaic in ArcGIS. All eight soil characteristics were retrieved with a spatial resolution of 250 × 250 m. The climatic variables were prepared based on data taken from the synoptic meteorological stations, https://data.noaa.gov and https://www.worldclim.org websites. These variables were interpolated using of a Kriging method and mapped by ArcGIS 10.7. In this study, we used the land use and lithology maps generated by the Iran Forest, Rangeland and Watershed management Organization and the Geological Survey and Mineral Exploration of Iran, respectively. The original spatial resolution for the landuse and lithology maps were 250 m × 250 m. Generally, all layers were converted to a consistent spatial resolution of 150 × 150 m.

**Table 1:** List of factors potentially controlling soil salinity.

No	Factor controlling soil salinity	No	Factor controlling soil salinity
1	Aspect	25	OCD
2	BI	26	Plan curvature
3	Bulk density	27	Precipitation
4	CRSI	28	Profile curvature
5	CEC	29	SI
6	Clay content	30	SI1
7	Clay index	31	SI2
8	Coarse fragment	32	SI3
9	Curvature	33	SIB
10	DEM	34	SRI
11	EVI	35	Sand content
12	Evaporation	36	SAVI
13	ENDVI	37	Silt content
14	Flow direction	38	Slope
15	FSEN	39	TRI
16	GFV	40	TSC
17	Lithology	41	TST
18	GVMI	42	TPI
19	Gypsum index	43	TWI
20	Landuse	44	TEVI
21	LSF	45	VD
22	MNDWI	46	WVIVI
23	NDVI	47	WVWI
24	NDI		

### Salinity inventory map

**Figure 2:**
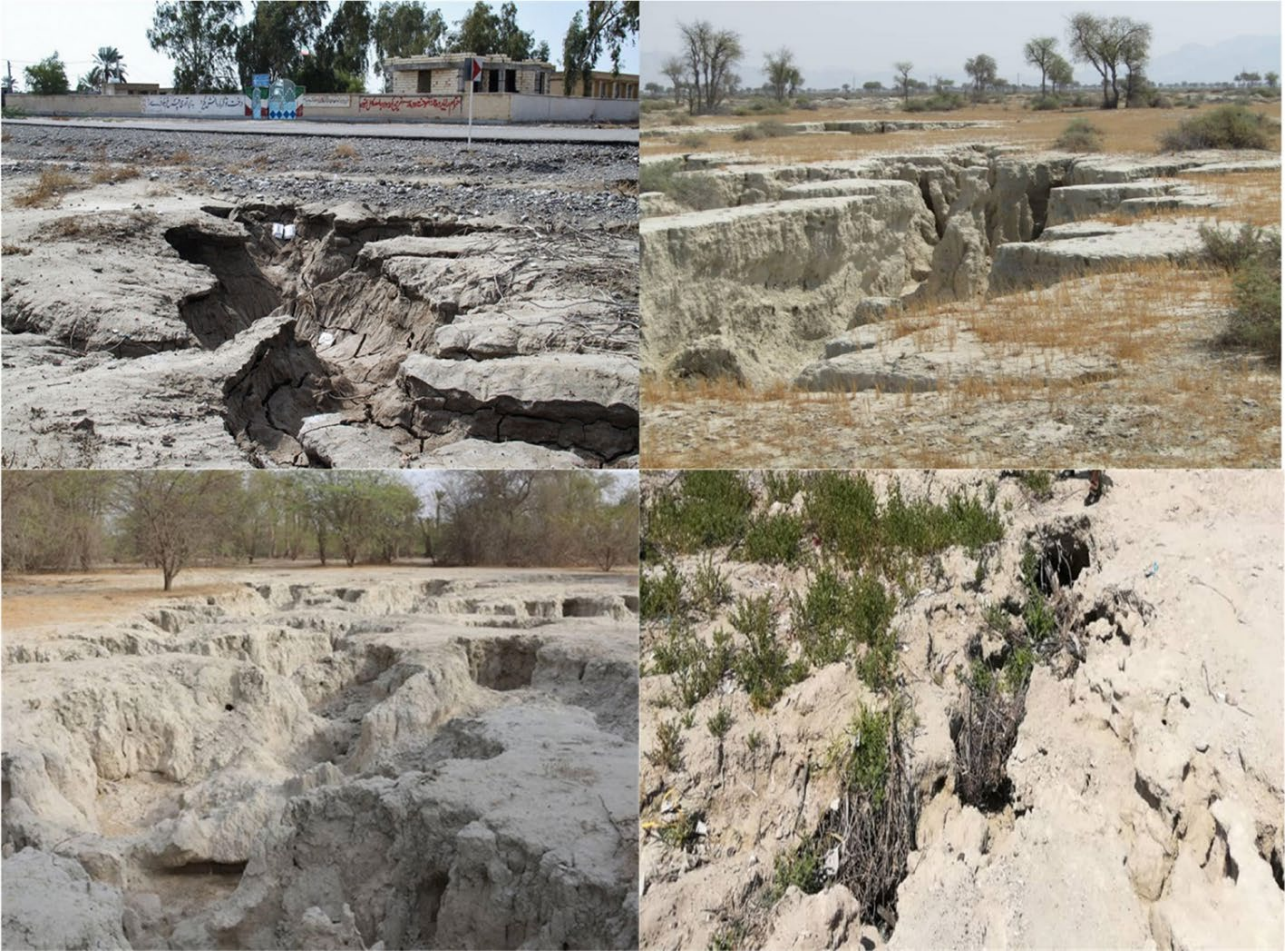
Photographs showing the land surface in the study area.

For developing the mapped inventory of soil salinity in the study area, we measured electrical conductivity (ECe) from the saturated extraction in the < 2 mm fraction in 476 samples collected from surface soils^18^. The soil samples (with 1 kg weight and 0–20 cm depth)^7^ with a good spatial distribution at the locations with the flat and plain topography were collected during the September 2018 to September 2019 in the study area (Fig. 2). For constructing DL predictive models of soil salinity, 333 (70%) and 143 (30) samples were selected randomly as training and validation datasets, respectively (Fig. 1b). We have used the Kfold method—as a typical cross validation method—to evaluating the performance of models. The spatial maps for 18 effective factors controlling soil salinity are presented in S1 and S2.

### Factor selection using stepwise forward regression (SFR)

Stepwise regression (SR) uses an automatic process for choosing predictive variables^19,20^. Here, the main approaches include forward selection, backward elimination and bidirectional elimination. In this study, we used stepwise forward regression (SFR) for factor selection. SFR—a stepwise regression (SR) approach—starts from the null model and adds a variable that improves the model the most, one at a time, until the specified criteria are met. The F-statistic and corresponding p-value are criteria for entering predictor variables into the model. The most important factors are chosen so that the model: (1) has the smallest p-value; (2) provides the maximum increase in R^2^, or; (3) provides the greatest reduction in the sum of residuals squared of the model.

### DL models for the spatial mapping of soil salinity

#### Deep Boltzmann machine (DBM)

Boltzmann machines (BMs)^21^ comprise a network of symmetrically coupled stochastic binary units consisting of a set of **v** ϵ {0, 1}^D^ (as visible units), and **h** ϵ {0, 1}^P^ (as a set of hidden units)^22^. In comparison with other BMs such as fully connected BMs, DBM are interesting for the three following reasons: (1) due to potential for learning internal representations that become increasingly complex; (2) high-level representations can be built from a large supply of unlabelled sensory inputs and very limited labelled data can then be used to fine-tune the model for a specific task in hand, and; (3) due to incorporating top-down feedback, allowing DBMs to better propagate uncertainty associated with ambiguous inputs^22^. In this study, this specific model included three layers consisting of a visible layer, three hidden layers (details are presented in Table 2) and a linear regression layer. For optimizing parameters, we applied a grid search automatic optimization algorithm in python.

**Table 2:** Details of the hyper-parameters of the DBM.

Parameter	Value
rbm1.learning_rate	0.06
rbm1.n_iter	10
rbm1.n_components	10
rbm2.learning_rate	0.06
rbm2.n_iter	10
rbm2.n_components	10
rbm3.learning_rate	0.06
rbm3.n_iter	10
rbm3.n_components	10

DBM learns the factors from the raw data and the factors extracted in one layer are applied as hidden variables as input to the subsequent layer. DBM incorporates a Markov random field for layer-wise data pre-training and then provides feedback from the upper layer to the backward layers^23^. By applying the backpropagation technique, the training algorithm is fine-tuned. The energy of the state {**v, h**} can be described as follows^24^:

1 $$E \left( {{\varvec{v}}, {\varvec{h}}; \theta } \right) = - {\varvec{v}}^{T} {\varvec{W}}^{1} {\varvec{h}}^{1} - {\varvec{h}}^{1T} {\varvec{W}}^{2} {\varvec{h}}^{2} - {\varvec{h}}^{2T} {\varvec{W}}^{3} {\varvec{h}}^{3} ,$$

where h = {**h**^1^, **h**^2^, **h**^3^} and θ = {**W**^1^, **W**^2^, **W**^3^} indicate the set of hidden units and the model parameters, representing visible-to-hidden and hidden-to-hidden symmetric interaction terms, respectively. The probability that the model assigns to a visible vector **v** is expressed as:

2 $$P \left( {{\varvec{v}}; \theta } \right) = \frac{{P \left( {{\varvec{v}}; \theta } \right)}}{Z\left( \theta \right)} = \frac{1}{Z \left( \theta \right)} \mathop \sum \limits_{{\varvec{h}}} \exp \left( { - E \left( {{\varvec{v}}, {\varvec{h}}^{1} , {\varvec{h}}^{2} , {\varvec{h}}^{3} ; \theta } \right)} \right).$$

**Figure 3:**
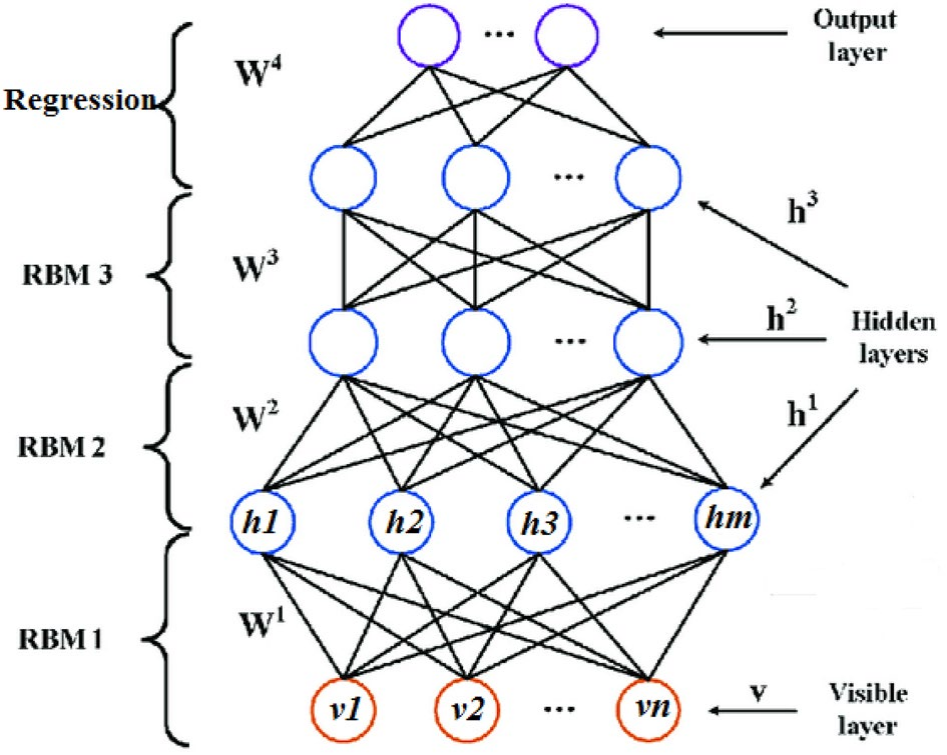
Conceptual diagram of the DBM.

The derivative of the log-likelihood with respect to parameter vector W^1^ takes the following form:

3 $$\frac{{\partial log P \left( {{\varvec{v}}; \theta } \right)}}{{\partial W^{1} }} = E_{{P_{data} }} \left[ {{\varvec{vh}}^{{1^{T} }} } \right] - E_{{P_{model} }} \left[ {{\varvec{vh}}^{{1^{T} }} } \right]$$

where $E_{{P_{data} }} \left[ . \right]$ indicates an expectation with respect to the completed data distribution $P_{data} \left( {{\varvec{h}}, {\varvec{v}}; \theta } \right)$ = P $\left( {{\varvec{h}}{|}{\varvec{v}};\theta } \right)$ $P_{data} \left( {\varvec{v}} \right), {\text{with}} P_{data} \left( {\varvec{v}} \right) = \frac{1}{N} \mathop \sum \limits_{n} \delta \left( {{\varvec{v}} - {\varvec{v}}_{n} } \right)$ indicate the empirical distribution, and $E_{{P_{model} }} \left[ . \right]$ indicates an expectation with respect to the distribution defined by the model. The derivatives with respect to parameters W^2^ and W^3^ take similar forms but instead involve the cross-products **h**^1^ **h**^2^ and **h**^2^ **h**^3^, respectively. The structure of DBM network is presented in Fig. 3.

#### One dimensional convolutional neural networks (1DCNNs)—long short-term memory (LSTM) hybrid deep learning (DL) model (1DCNN-LSTM hybrid DL model)

CNN—is a special type of NN (neural network)—used widely in the image processing field. In CNN, a map of factors is applied for extracting factors from the input of the previous layer with a convolution operation. CNN consists of three layers comprising pooling, convolutional and fully connected^10^. The pooling layer, by reducing the size of the output from a stack layer to the next and at the same time preserving important information, can reduce the complexity of the calculations. The convolutional layer provides the outputs of the pooling layer and maps them to the next layer. The last layer for data classification is fully connected^25,26^.

CNN networks include one-dimensional CNN (1DCNN), two-dimensional CNN (2DCNN), and three-dimensional CNN (3DCNN) networks^27^. CNN has commonly been used with 2D data (e.g., images)^28^. A 1DCNN is a modified version of 2DCNN^29,30^. In comparison with 2DCNN, 1DCNN has the following advantages: (1) high computational simplicity; (2) suitability for real-time and low-cost applications, and; (3) capacity to learn challenging tasks.

RNNs—a DL model were developed in the 1980s as an extension of conventional feedforward NN^31^. RNNs have three essential elements comprising input (x_0,_ x_1,_ …, x_i,_ x_i+1,_ …), hidden (h_0,_ h_1,_ …, h_i,_ h_i+1,_ …) and output (y_0,_ y_1,_ …, y_i,_ y_i+1,_ …)^32^. LSTM—suggested by Hochreiter and Schmidhuber^33^,—is a special kind of structure of deep RNNs. In comparison with the traditional RNNs, LSTM has benefit of the forget gate^34^. Therefore, by adding a self-loop technique to produce a long-term flow path, LSTM solves a problem faced by traditional RNNs. A unit of LSTM has three control gates consisting of: (1) input (*i*), (2) output (*o*) and (3) forget (*f*). A LSTM unit can expressed as follows:

4 $$ft = \sigma \left( {W^{(f)} {\text{x}}_{t} + U^{(f)} h_{t - 1} } \right)$$

5 $$i_{t} = \sigma_{{}} \left( W \right)^{(i)} {\text{x}}_{t} + U^{(i)} \left. {h_{t - 1} } \right)$$

6 $$o_{t} = \sigma (W^{{(o)}} {\text{x}}_{t} + U^{{(o)}} \left. {h_{{t - 1}} } \right)$$

7 $$ci_{t} = \sigma _{h} \left( {W^{{(ci)}} {\text{x}}_{t} + U^{{(ci)}} h_{{t - 1}} } \right)$$

8 $$c_{t} = f_{{\text{t}}} {\text{o c}}_{{{\text{t}} - {1}}} + i_{t} o \, ci_{t}$$

9 $$h_{t} = o_{{\text{t}}} {\text{o }}\sigma_{{\text{h}}} (c_{t} )$$

where *o*, *t,* . *f*_*t*_ ∈ *R*^*h*^*, i*_*t*_ ∈ *R*^*h*^ and *o*_*t*_ ∈ *R*^*h*^ are the element-wise product, the time step, the activation vectors for the forget, input and output gates, respectively. The initial values for *c*_*0*_ and *h*_*0*_ are zero. x_t_ ∈ *R*^*d*^*, h*_*t*_ ∈ *R*^*h*^*, ci*_*t*_ and *c*_*t*_ indicate the input vector, output vector, the cell input activation vector and the cell state vector, respectively. W ∈ R^h×d^, U ∈ R^h×h^ and b ∈ *R*^*h*^ represent the weight metrics and bias vector parameters, respectively. *σ* and *σ*_*h*_ are sigmoid and hyperbolic tangent functions (*σ*_*h*_ (*x*) = *x*), respectively.

**Figure 4:**
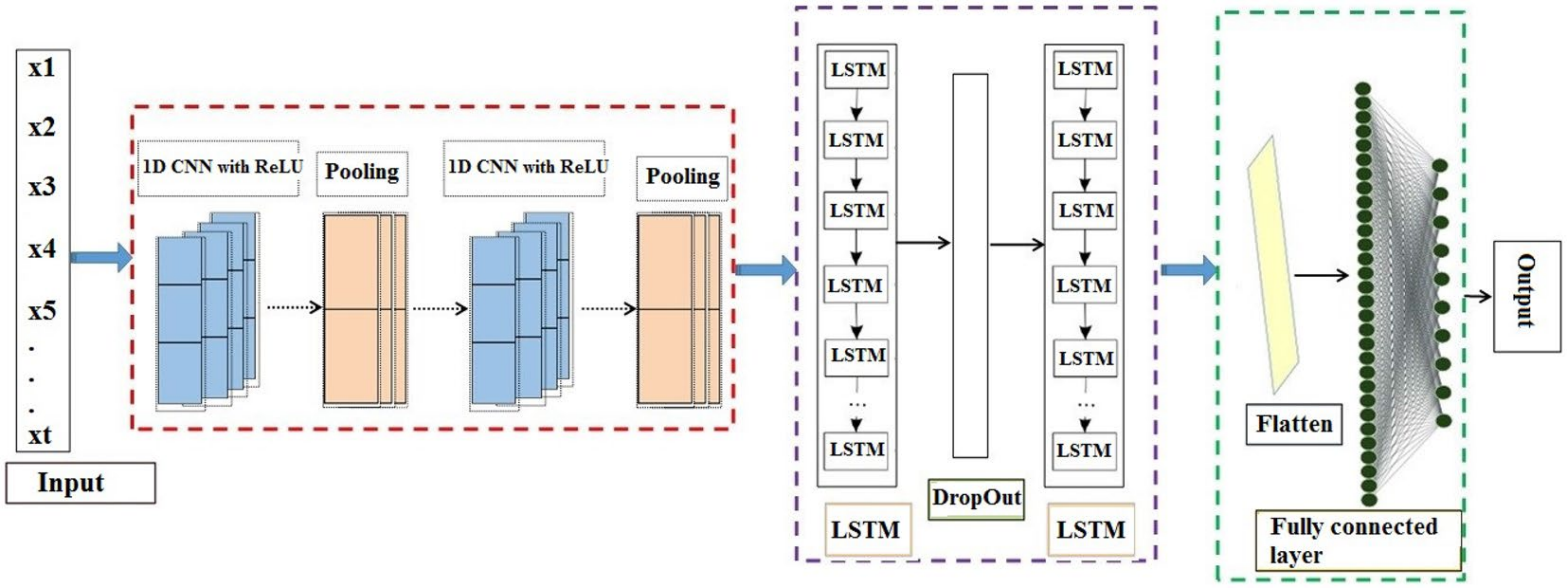
Conceptual diagram of the 1DCNN-LSTM model.

Here, we applied a 1DCNN-LSTM hybrid DL model for mapping soil salinity in the study area. The structure of the 1DCNN-LSTM hybrid model consisted of nine layers including a convolution 1D layer (with input_dim = 18, number of neurons = 128, and ReLU (rectifier linear unit) activation activation), max-pooling layer, convolution 1D layer (with neurons = 64, and ReLU activation function), max-pooling layer, LSTM layer with number of neurons = 32, dropout layer with a value = 0.25, LSTM layer with number of neurons = 16, flatten layer, and fully connected layer with neuron = 1. In the compiling stage of the model, mean square error was chosen as the loss function. RMSProp was selected as the optimizer. In the fit stage of the model, the number of epochs (100, 200 and 500) and batch sizes (10, 15 and 20) were selected as 500 and 10, respectively, because these values delivered the lowest error. The conceptual diagram for the 1DCNN-LSTM hybrid model is presented in Fig. 4.

### Interpretability of DL models using game theory

Game theory^35^ using two measures consisting of the permutation feature importance measure (PFIM)^36^ and Shapely Additive exPlanations (SHAP) was used to determine the contributions of individual variables to predictive model performance in the Minab plain. PFIM measures the importance of a feature by calculating the improvement in the model prediction error.

SHAP specifies the explanation as follows:

10 $$g\left( {{{{\rm z}^{\prime}}}} \right) = \emptyset _{0} + ~\mathop \sum \limits_{{j = 1}}^{M} \emptyset _{j} ~{{\rm z}}_{j} ^{\prime } ~$$

where *g*, $z^{\prime}$ ∈ {0,1}^*M*^, and *M* indicate the explanation model, the coalition vector and the maximum coalition size, respectively.

### Quantifying uncertainty associated with the DBM and 1DCNN-LSTM hybrid models using DeepQuantreg

Quantile regression (QR)—suggested by Koenker and Bassett^37^,—is a typical least-square approach in linear regression. Here, DeepQuantreg^38^ was applied to quantify uncertainty associated with the two DL models. The input layer is the covariate vector *x*_*j*_ (*j* = 1, 2, …, *J*). The hidden layers are dense, i.e. fully connected by the nodes. The output layer is given by applying the activation function to the inner product between the input and the hidden-layer weights plus the hidden-layer bias. For example, assuming there are two hidden layers and *J* input variables, the output of the *k*th hidden node for the first hidden layer (*g*_*k*_) can be expressed as follows:

11 $$g_{k} = f_{1} \left( {\mathop \sum \limits_{j = 1}^{J} x_{j} w_{jk}^{\left( h \right)} + b_{k}^{\left( h \right)} } \right), k = 1, 2, \ldots , K,$$

The output of the *l*th hidden node in the second hidden layer (*h*_*l*_) can expressed as follows:

12 $$h_{l} = f_{2} \left( {\mathop \sum \limits_{k = 1}^{K} g_{k} w _{kl}^{\left( h \right)} + b_{l}^{\left( h \right)} } \right), l = 1, 2, \ldots ,L,$$

where, *f*_1_ (.), *f*_2_ (.), *w*^(h)^ and *b*^(h)^ indicate activation functions for the first and second hidden layers, the hidden layer weights and bias, respectively.

The output layer with a linear activation function gives the estimate of the conditional $\tau$th quantile ($Q_{i}^{\left( \tau \right)} )$ for the *i*th subject as;

13 $$Q_{i}^{\left( \tau \right)} = \mathop \sum \limits_{l = 1}^{L} h_{l} w_{l}^{\left( o \right)} + b^{\left( o \right)}$$

where *w*^*(o)*^ indicates the output layer weights, and *b*^*(o)*^ is the bias.

### Assessment of the performance of the DBM and 1DCNN-LSTM hybrid models for generating spatial maps of soil salinity

**Figure 5:**
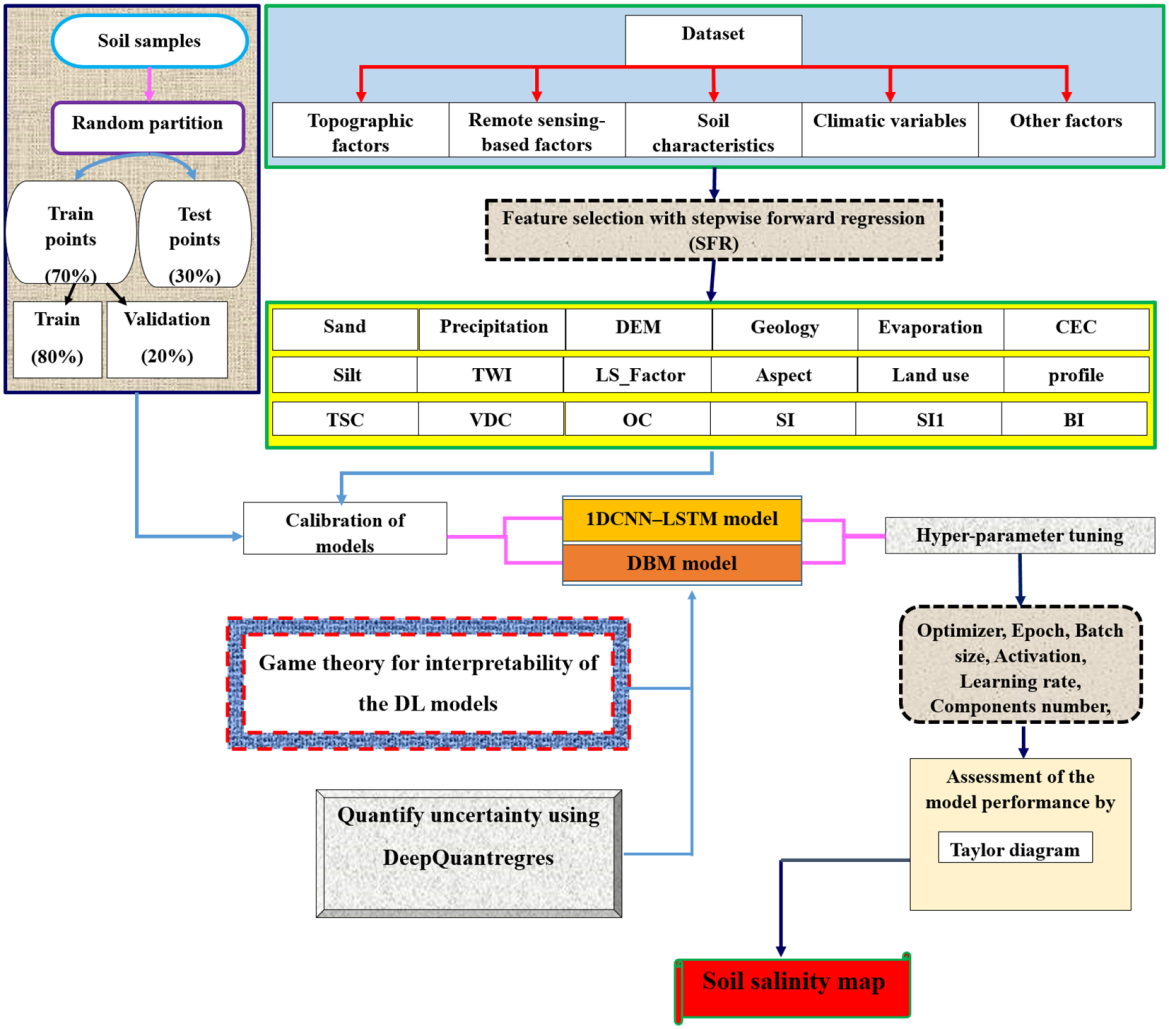
Flowchart of the methodology used to map soil salinity in the study area.

Evaluating the accuracy of the spatial maps of environmental hazards generated by predictive models is essential for selecting the most efficient predictions. Thus, a Taylor diagram^39^ was used to assess the performance of the two DL models for both the training and validation datasets. The key methodological steps used in this study are summarised in Fig. 5.

## Results and discussion

### Factors selected as input to the DL models

As shown in Table 3, 18 factors consisting of sand content, precipitation, vertical distance to channel (VDC), lithology, evaporation, CEC, silt content, topographic wetness index (TWI), LS factor, salinity index (SI), brightness, salinity index (SI1), terrain surface convexity (TSC), DEM, organic carbon content (OCC), aspect, profile and landuse were selected as the effective controls on soil salinity in the study area. The lowest and highest values of RMSE were estimated for land use (RMSE = 1.2 and R^2^ = 0.94) and sand content (RMSE = 2.3 and R^2^ = 0.77), respectively. Overall, among the effective factors, the strongest controls were related to the soil, terrain and topographic characteristics. Similarly, Mohammadifar et al.^10^, reported that the topographic-based factors (TPI, TWI and flatness), vegetation and soil characteristics (e.g., saturate percent, carbonate index and salinity index (SI1)), are the effective factors controlling soil salinity in an arid catchment in southern Iran. Previous work^7^ reported that TWI and flatness are the two most important factors controlling soil salinity in a region in China.

**Table 3:** The results of controlling factor selection by SFR.

Step	Variable entered	R^2^	c(p)	AIC	RMSE
1	Sand content	0.77	810.5	1493	2.3
2	Precipitation	0.86	365	1329	1.8
3	VDC	0.9	196.6	1237	1.5
4	Geology	0.91	90.3	1161	1.4
5	Evaporation	0.92	77.7	1151	1.3
6	CEC	0.92	67.2	1142	1.3
7	Silt content	0.93	51.6	1129	1.3
8	TWI	0.93	44.5	1123	1.3
9	LS factor	0.93	37.6	1116	1.3
10	SI	0.93	32	1111	1.3
11	Brightness index	0.93	18	1099	1.2
12	SI1	0.94	17	1098	1.2
13	TSC	0.94	16.4	1097	1.2
14	DEM	0.94	16.2	1097	1.2
15	OCC	0.94	16.2	1097	1.2
16	Aspect	0.94	16.5	1097	1.2
17	Profile	0.94	17	1098	1.2
18	Landuse	0.94	17.7	1098	1.2

### Spatial maps of soil salinity generated by DBM model and 1DCNN-LSTM hybrid model

**Figure 6:**
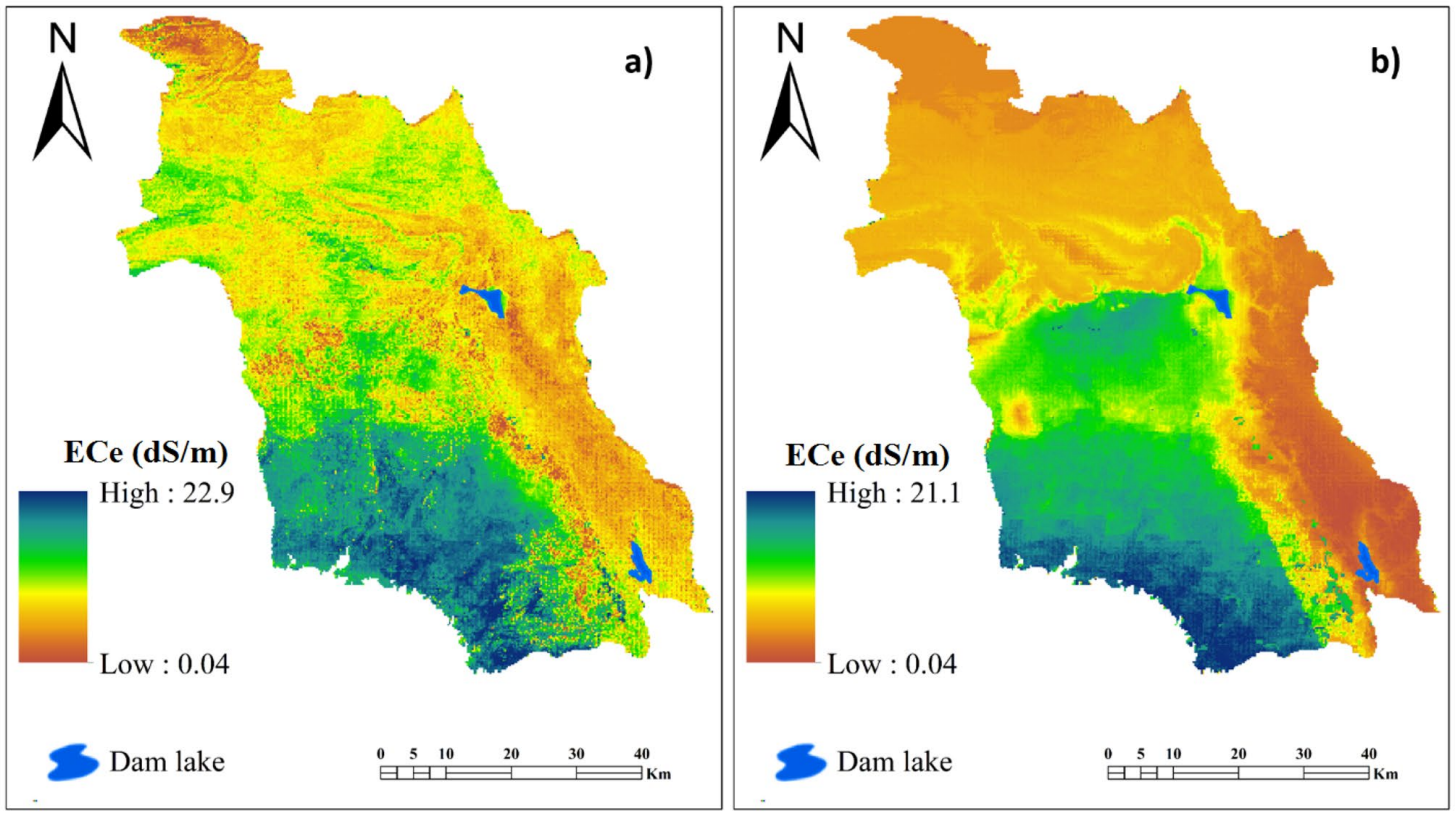
Spatial maps of soil salinity produced by: (**a**) DBM, and; (**b**) the 1DCNN-LSTM hybrid model.

The spatial maps for soil salinity in the Minab plain generated using the two DL models are presented in Fig. 6. Based on the DBM model (Fig. 6a), the lowest and highest values for ECe in the study area were 0.04 and 22.9 dS/m. The EC value predicted by the 1DCNN-LSTM hybrid model ranged between 0.04 and 21.1 dS/m (Fig. 6b). The values of EC predicted by both models are similar. Based on both models, the southern and southwestern parts of the study area with flat topography, lowland and agricultural land use are more susceptible to soil salinization. The lowest values of EC estimated in the eastern and northern parts of the study area coincide with highland landscapes and mountainous terrain. These patterns are consistent with those reported by^7,10^. Due to intensified drought over the past two decades, reductions in the groundwater table due to extraction for agricultural uses, and proximity of the western and southern parts of the study area to the coast of the Persian Gulf, and concomitant invasion of sea water into coastal aquifers, soil salinity has increased in these areas.

### Quantifying uncertainty associated with the DL models using DeepQuantreg

**Figure 7:**
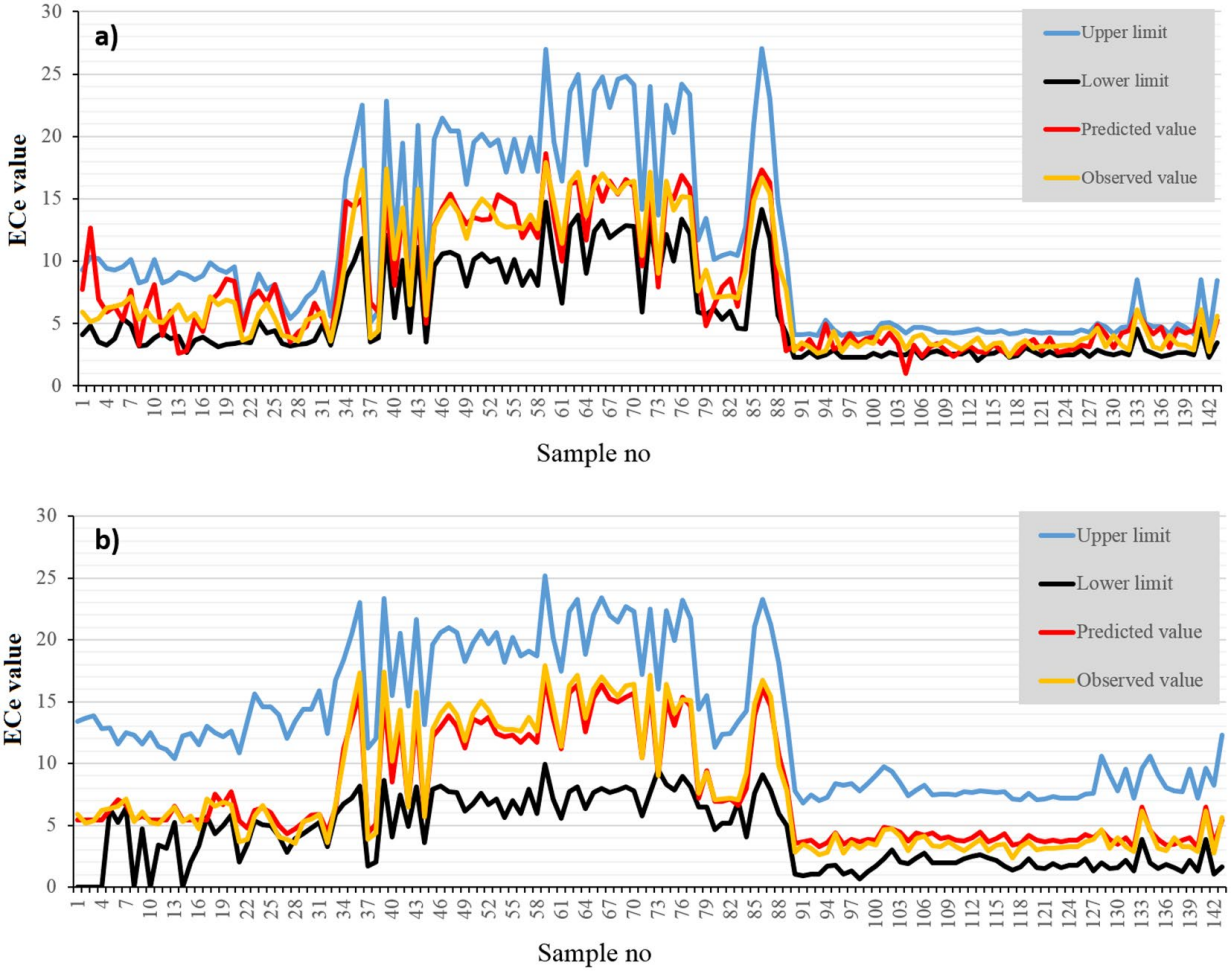
The uncertainty associated with the (**a**) DBM model and (**b**) 1DCNN-LSTM hybrid model estimated using DeepQuantreg.

The uncertainty associated with the soil salinity predicted by the two DL models using DeepQuantreg with 95% confidence limits is presented in Fig. 7. The values of ECe measured in the study area range between 2.4 and 17.9 dS/m, while those predicted by the DBM and 1DCNN-LSTM hybrid models ranged between 1 and 18.6 dS/m; and 3.2 to 16.8 dS/m, respectively. Based on the uncertainty associated with the predictions of the DBM model (Fig. 7a), the values of ECe in the study area ranged between 2 dS/m for sample 113 (the lower limit) to 27 dS/m for sample 86 (the upper limit). The uncertainty associated with the results of the 1DCNN-LSTM hybrid model (Fig. 7b) ranged between 0 (for samples 1–4, 8, 10, and 14) and 25 dS/m (for sample 59), respectively. Overall, the values of ECe predicted by both DL models and the corresponding uncertainty ranges estimated using DeepQuantreg were similar and in good agreement with the measured values.

### Assessment of the performance of the DL models

**Figure 8:**
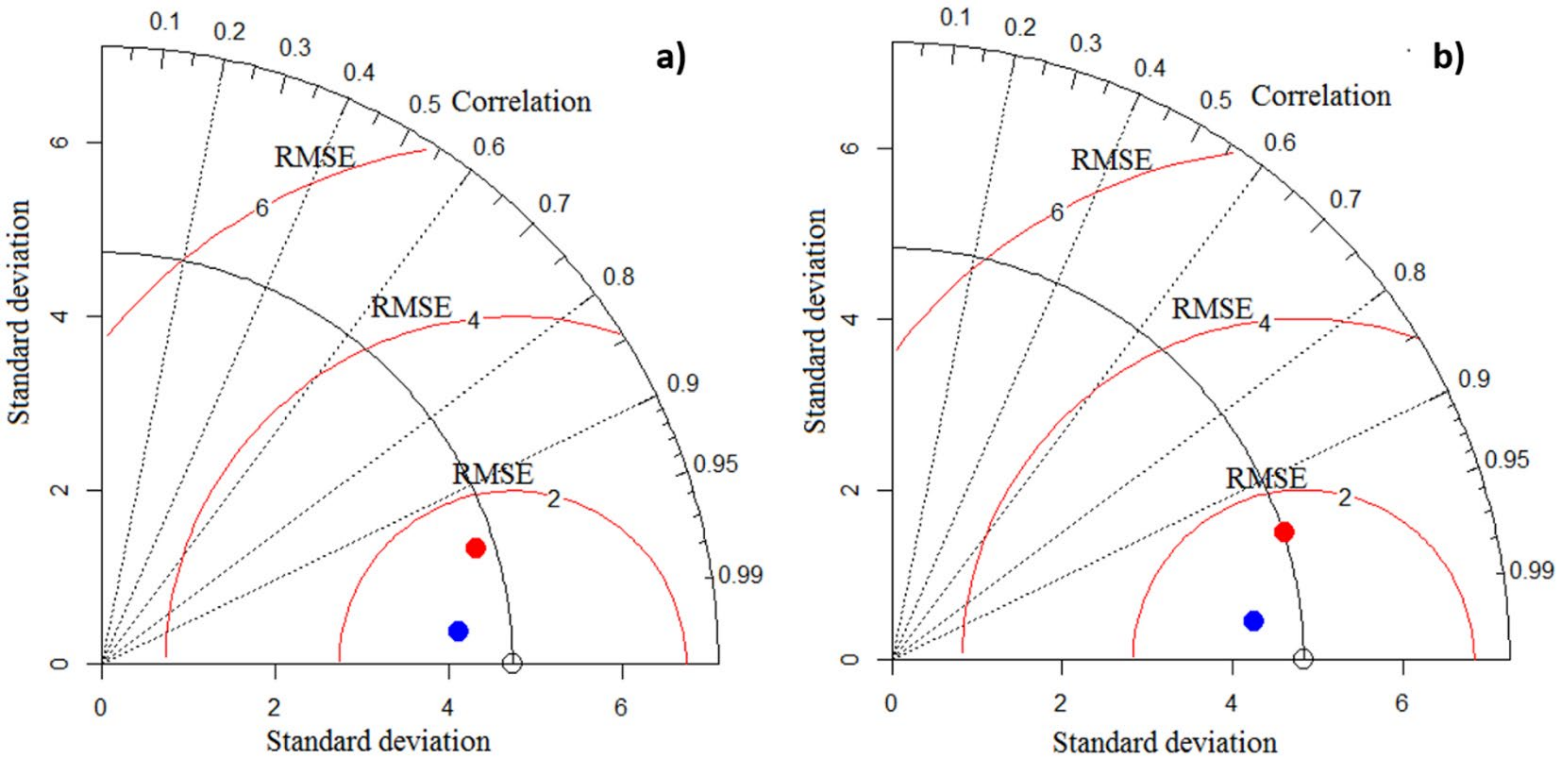
Taylor diagrams for assessment of DL model performance based on: (**a**) the training dataset, and; (**b**) the validation dataset. The red and blue points indicate the DBM and 1DCNN-LSTM hybrid model, respectively.

Taylor diagrams are presented in Fig. 8. Both models generated RMSE < 20%, indicating good performance for mapping soil salinity. The 1DCNN-LSTM hybrid model performed slightly better than the DBM model for both the training and the validation datasets. Hybrid models can improve the accuracy and performance of predictions^40^. Generally speaking, and in comparison with individual DL models, hybrid DL models are typically more efficient for prediction purposes^41–43^. DL models (e.g., deep convolutional neural networks (DCNNs), dense connected deep neural networks (DenseDNNs), recurrent neural networks-long short-term memory (RNN-LSTM) and recurrent neural networks-gated recurrent unit (RNN-GRU)) performed better than shallow ML models (e.g., bagged classification and regression tree (BCART), cforest, cubist, quantile regression with LASSO penalty (QR-LASSO), ridge regression and support vectore machine (SVM)) for production of the soil salinity spatial maps, and therefore we recommend applying DL models for prediction purposes in environmental sciences^10^.

### Relative importance of factors controlling soil salinity and their contributions to the model’s performance

**Figure 9:**
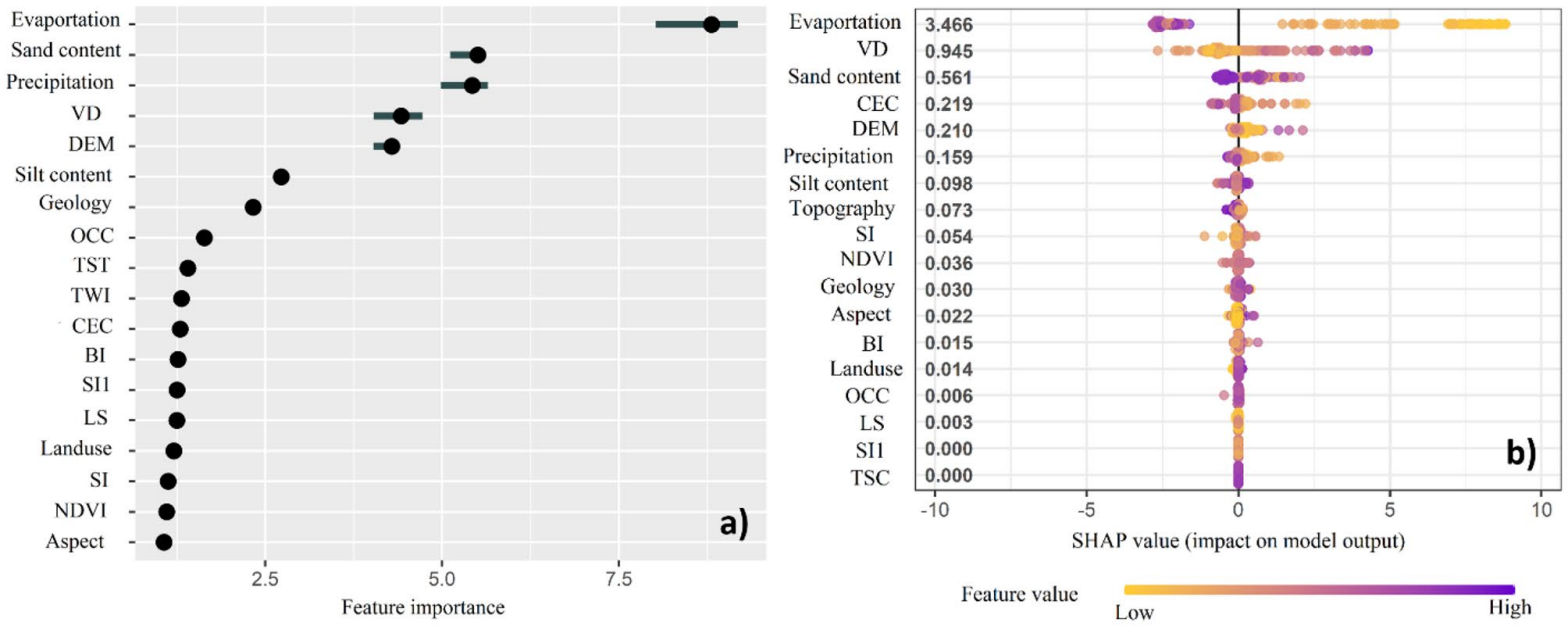
(**a**) The relative importance of factors controlling soil salinity determined by: (**a**) PFIM, and; (**b**) SHAP.

Based on the Fig. 9, among the 18 effective factors controlling soil salinity, four factors comprising evaporation, sand content, precipitation and vertical distance to channel were selected as the most important controls. Overall, the results provided by PFIM are consistence with those obtained from the SFR (Table 3). Based on the SHAP values, five factors (evaporation, vertical distance to channel (VD), sand content, CEC and DEM) have the highest impact on model performance. We conclude that overall, game theory is a valuable technique for assessing the interpretability of predictive models because this theory through SHAP and PFIM addresses the important concerns regarding the interpretability of more complex DM models.

## Conclusions

Our study delivers three contributions to existing understanding and international literature: (1) demonstration of factor selection by stepwise forward regression (SFR); (2) explicit assessment of the uncertainty associated with two DL models used to map soil salinity in the Minab catchment using deep quantile regression—DeepQuantreg, and; (3) evaluation of the interpretability of the predictive DL models using two measures (PFIM and SHAP) applied in game theory. Among the preliminary 47 factors potentially controlling soil salinity, 18 were selected as effective factors for mapping soil salinity. The 1DCNN-LSTM hybrid model performed slightly better than DBM model with respect to map soil salinity. The uncertainty ranges associated with soil salinity predicted by the 1DCNN-LSTM hybrid model estimated using DeepQuantreg are close to those corresponding values predicted using the DBM model. Overall, our work herein demonstrates that application of DL models in combination with explicit analysis of uncertainty and of interpretability using game theory can be useful for mapping spatial patterns in soil degradation.

## Supplementary Information

Supplementary Information.

## Data Availability

The datasets used and/or analysed during the current study available from the corresponding author on reasonable request.
