# Supplementary material for: Assessment of the uncertainty and interpretability of deep learning models for mapping soil salinity using DeepQuantreg and game theory
Source: Sci Rep. 2022 Sep 7;12:15167. doi: 10.1038/s41598-022-19357-4 (PMC9452570; doi:10.1038/s41598-022-19357-4)
Supplement: Supplementary file 1 — Supplementary Information. [file 41598_2022_19357_MOESM1_ESM.docx]

**
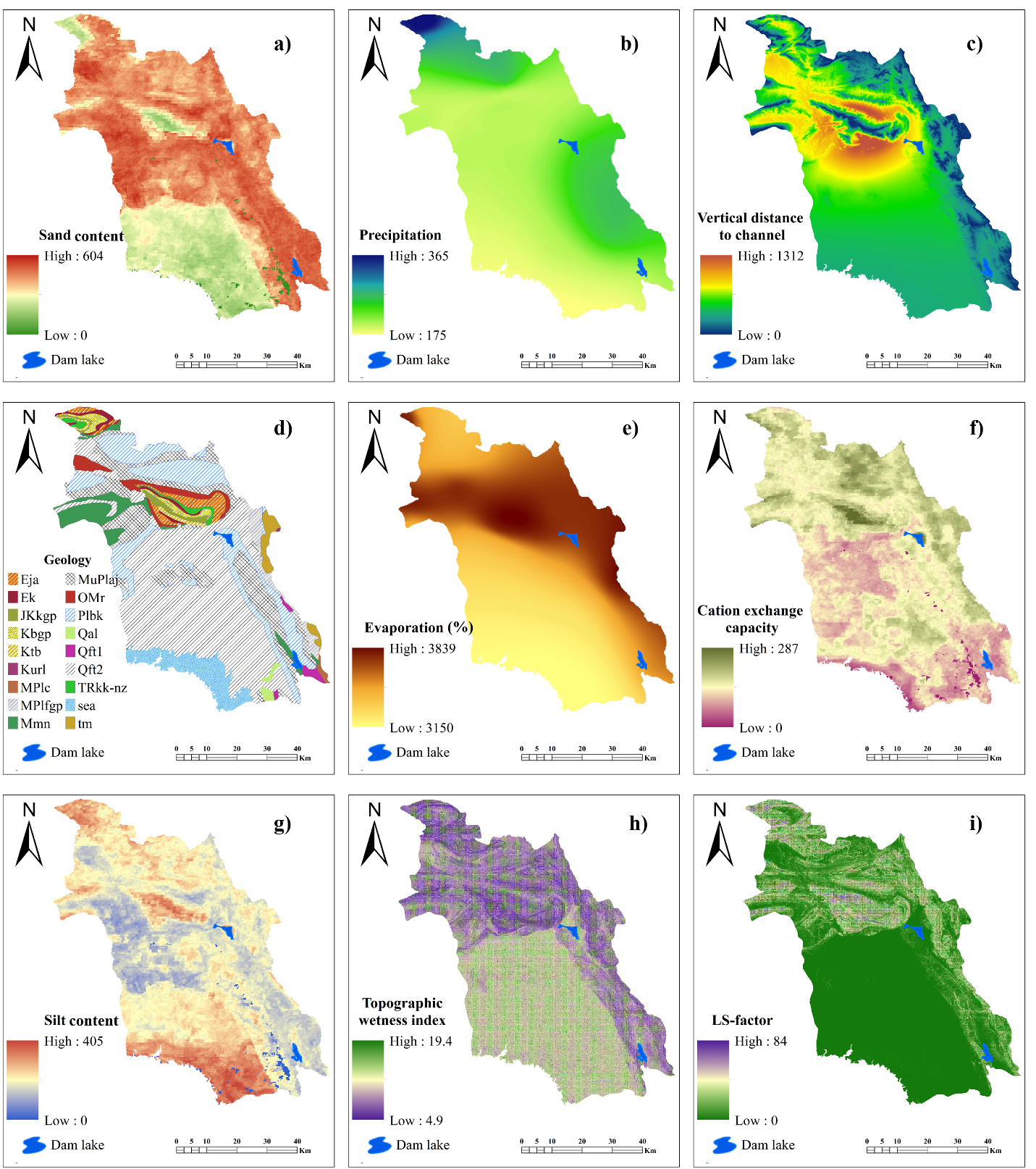
**

S1: Spatial maps for some effective factors controlling soil salinity: a) sand content, b) precipitation, c) vertical distance to channel, d) geology, e) evaporation, f) cation exchange capacity, g) silt content, h) topographic wetness index, and i) LS factor.


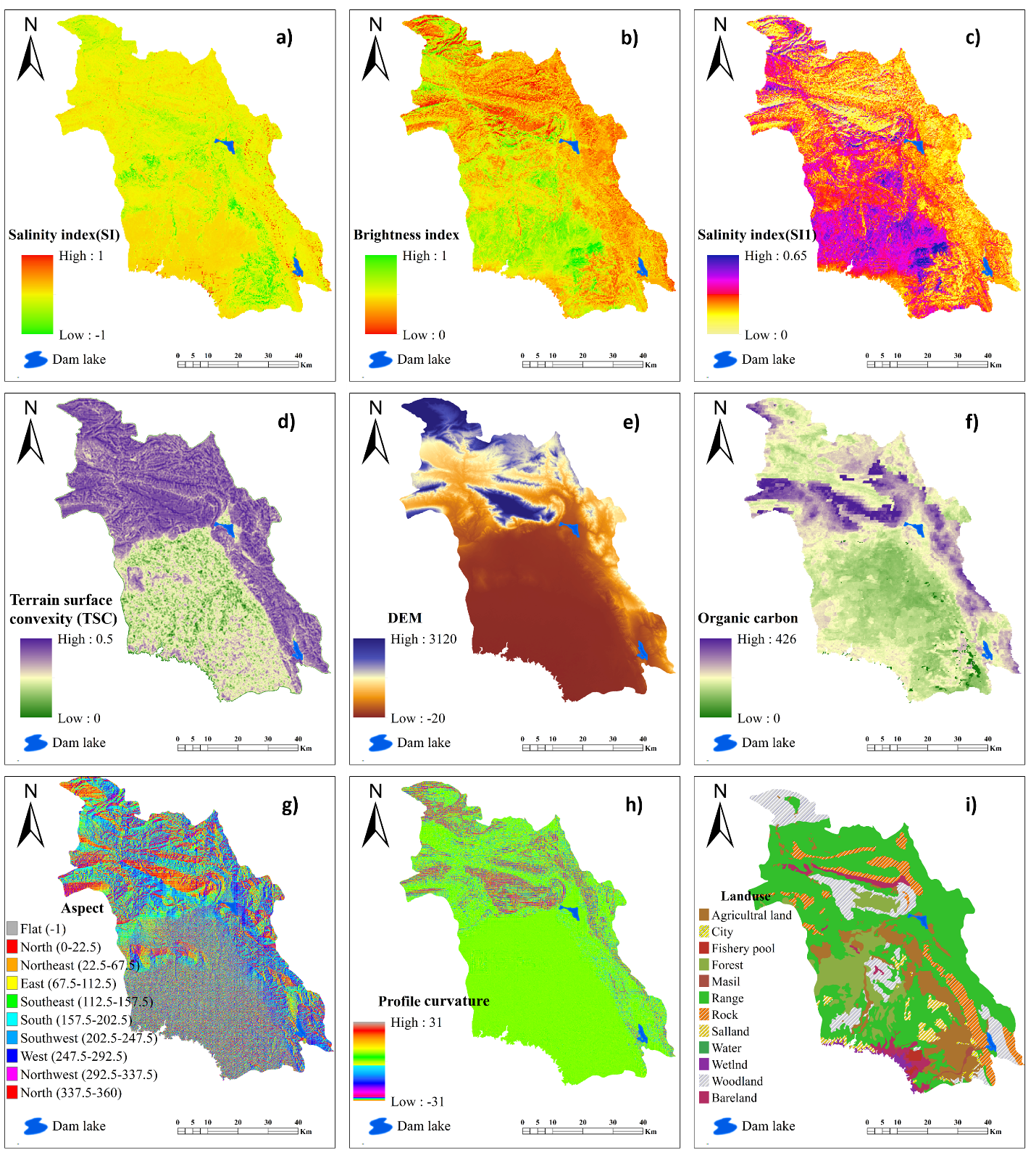


S2: Spatial maps for some effective factors controlling soil salinity: a) salinity index (SI), b) brightness index, c) salinity index (SI1), d) terrain surface convexity (TSC), e) DEM, f) organic carbon content, g) aspect, h) profile curvature, and i) land use.
